# Minimizing the evidence-practice gap – a prospective cohort study incorporating balance training into pulmonary rehabilitation for individuals with chronic obstructive pulmonary disease

**DOI:** 10.1186/s12890-015-0067-2

**Published:** 2015-07-23

**Authors:** Samantha L. Harrison, Marla K. Beauchamp, Kathryn Sibley, Tamara Araujo, Julia Romano, Roger S. Goldstein, Dina Brooks

**Affiliations:** Department of Respiratory Medicine, West Park Healthcare Centre, Toronto, ON Canada; Department of Physical Medicine and Rehabilitation, Spaulding Rehabilitation Hospital, Harvard Medical School, Cambridge, MA USA; Centre for Healthcare Innovation and Department of Community Health Sciences, University of Manitoba, Winnipeg, MB Canada; Department of Physical Therapy, University of Toronto, Toronto, ON Canada; Department of Medicine, University of Toronto, Toronto, ON Canada

**Keywords:** Knowledge translation, Knowledge to action, COPD, Balance, Falls, Pulmonary rehabilitation, Physiotherapists

## Abstract

**Background:**

We have recently demonstrated the efficacy of balance training in addition to Pulmonary Rehabilitation (PR) at improving measures of balance associated with an increased risk of falls in individuals with Chronic Obstructive Pulmonary Disease (COPD). Few knowledge translation (KT) projects have been conducted in rehabilitation settings. The goal of this study was to translate lessons learnt from efficacy studies of balance training into a sustainable clinical service.

**Methods:**

Health care professionals (HCPs) responsible for delivering PR were given an hour of instruction on the principles and practical application of balance training and the researchers offered advice regarding; prescription, progression and practical demonstrations during the first week. Balance training was incorporated three times a week into conventional PR programs. Following the program, HCPs participated in a focus group exploring their experiences of delivering balance training alongside PR. Service users completed satisfaction surveys as well as standardized measures of balance control. At six month follow-up, the sustainability of balance training was explored.

**Results:**

HCPs considered the training to be effective at improving balance and the support provided by the researchers was viewed as helpful. HCPs identified a number of strategies to facilitate balance training within PR, including; training twice a week, incorporating an interval training program for everyone enrolled in PR, providing visual aids to training and promoting independence by; providing a set program, considering the environment and initiating a home-based exercise program early. Nineteen service users completed the balance training [ten male mean (SD) age 73 (6) y]. Sixteen patients (84 %) enjoyed balance training and reported that it helped them with everyday activities and 18 (95 %) indicated their wish to continue with it. Scores on balance measures improved following PR that included balance training (all p < 0.05). At six month follow-up balance training is being routinely assessed and delivered as part of standardised PR.

**Conclusions:**

Implementing balance training into PR programs, with support and training for HCPs, is feasible, effective and sustainable.

**Trail registration:**

Clinical Trials ID: NCT02080442 (05/03/2014)

**Electronic supplementary material:**

The online version of this article (doi:10.1186/s12890-015-0067-2) contains supplementary material, which is available to authorized users.

## Background

Chronic Obstructive Pulmonary Disease (COPD) is characterised by chronic airflow limitation which is not fully reversible. Over 10 % of the global population are affected by this condition with the prevalence continuing to rise. By 2020, COPD is set to become the third leading cause of death worldwide [1, 2]. Although the primary symptoms associated with COPD include shortness of breath on exertion, a chronic cough and frequent sputum production, secondary effects of the disease are increasingly recognized. Impairments in peripheral muscle function, mobility and exercise capacity are well established [3, 4] and the incidence of falls in COPD is four times that reported in a ‘healthy’ elderly population [5, 6]. As impaired balance is a major risk for falls [7–9], it is perhaps unsurprising that individuals with COPD exhibit important deficits in balance control [8, 10–14].

Pulmonary Rehabilitation (PR), consisting of exercise, education and psychological support, is recommended as a standard of care for patients with COPD [15, 16]. Despite recently documented deficits in balance control, balance training and fall prevention strategies are not included in international guidelines for PR and very few programs include any standardized balance measurement [17–19]. Given the recommendation that exercise be combined with balance training to reduce falls in older adults who are at risk [7], PR appears to be an ideal setting in which to implement a balance training program.

West Park Healthcare Centre (WPHC) has an internationally recognized reputation for respiratory research which is supported by the clinical services. Equally, the evidence gleaned from high quality research studies is made available to managers and clinicians, serving to inform clinical practice at WPHC [20, 21]. Over a period of five years the research team at WPHC has contributed significantly to the evidence exploring balance issues in individuals with COPD. We began by identifying balance impairment in patients with COPD and understanding the systems involved [8, 22]. Secondly, we highlighted the ineffectiveness of PR delivered in the absence of any specific balance training at improving balance control and confidence [23] and most recently we conducted a randomized controlled trial (RCT) to demonstrate the efficacy of balance training, in addition to PR, at improving balance performance in a group of patients with moderate to severe COPD [24].

Reducing the gap between evidence and practice is essential to ensure patients receive optimal care. Yet, few knowledge translation (KT) projects have been conducted involving HCPs, namely physiotherapists, working within rehabilitation settings [25, 26]. Therefore, the goal of this study was to translate lessons learnt from efficacy studies of balance training into a sustainable clinical service. Specifically, the study aim was to assess the feasibility of incorporating balance training as a component of PR programs for individuals with COPD. This information may serve to inform other HCPs how to best deliver balance training within PR.

In this manuscript we report how the researchers facilitated the incorporation of balance training into PR by communicating with the HCPs responsible for its delivery and by providing practical advice regarding; prescription, progression and practical demonstrations. We have described the development of the balance training program and the manner in which it was delivered in terms of; assessment, content and staffing required. The findings gleaned from a focus group conducted with the HCPs who delivered balance training within PR are described along with the results from satisfaction surveys completed by the service users. The effectiveness of the balance training on improving measures of balance control associated with a risk of falls and standard PR outcome measures is reported and compared with measures of the same balance intervention obtained during the efficacy study [24]. Finally, the status of balance training within PR six months following completion of the study is described.

## Methods

### Study design

This was a prospective, single group, longitudinal study design. Ethical approval for this study was obtained from The Joint Bridgepoint Health – West Park Healthcare Centre – Toronto Central Community Care Access Centre – Toronto Grace Health Centre Research Ethics Board, and all participants provided written informed consent prior to inclusion in the study.

### Participants

All the HCPs responsible for delivering the in-patient and out-patient PR programs at WPHC were invited to take part in the study.

Between May 2013 and January 2014, consecutive patients with stable COPD accepted for PR were approached. To be eligible for recruitment patients had to have; a self-reported decline in balance, or fall in the last five years, or a recent near fall and a smoking history greater than 10 pack years [24].

### Intervention: pulmonary rehabilitation with balance training

#### Pulmonary rehabilitation setting

PR is delivered both as a six week in-patient program and a 12 week out-patient program at WPHC. Both programs deliver exercise training three times a week with each session lasting one hour. All patients are required to complete the Six-Minute Walk Test (6MWT) [27] and the Chronic Respiratory Questionnaire-Self-reported (CRQ-SR) [28] pre and post-PR. The 6MWT is an assessment of functional exercise capacity and the CRQ-SR is a questionnaire reporting on patients’ health status. These are the only outcome measures routinely recorded as part of the PR programs administered at WPHC. Data pertaining to these measurements was collected from clinical records.

To enable the pooling of results from inpatient and outpatient programs all patients underwent balance training three times a week for a period of six weeks for a targeted total of 18 sessions, each one lasting 30 min. This protocol is in keeping with the approach from a previous RCT [24].

#### Support and training for HCPs

Before the commencement of balance training, the researchers communicated with the ‘clinical service lead’ to arrange a time when all the HCPs responsible for the PR program could attend a one hour training session. The hour comprised of a power-point presentation given by one of the researchers (SH) regarding the benefits and practical applications of balance training and familiarization with the balance equipment (i.e. ramp, foam, bosu ball, step, obstacle course). For one week, two researchers (SH and JR) assisted the HCPs with the balance training sessions delivering guidance on; prescription, progression and providing practical demonstrations as requested.

### Program delivery for balance training

#### Preparation

The program content was informed by previous research [8, 22–24] and conversations with the HCPs responsible for its delivery. Following the one hour education session, the HCPs decided how to best incorporate balance training into PR, through a series of meetings held independently of the researchers. The researcher’s (SH) role was to ensure the balance training was delivered in a way which was consistent with the evidence-base. For example, that the training was delivered thrice weekly, for 30 min per session and included all subcomponents of balance, especially biomechanics, transitions and gait, known to be the most impaired in individuals with COPD [22, 24]. Training logs, developed in collaboration between researchers and HCPs, contained three stages of balance training. Stage one contained basic balance exercises including; narrow stance with eyes closed, tandem stance, normal stance on foam, walking sideways. Stage two involved exercises such as; narrow stance throwing and catching a ball, narrow stance on foam with eyes closed, perturbations in narrow stance, sit on floor and stand up without a chair. Stage three was the most advanced requiring individuals to; stand on the bosu ball throwing and catching the ball, perform sit to stand with a medicine ball, respond to perturbations in tandem stance, complete a high level obstacle course. Decisions to adapt the PR program to accommodate balance training were made by the HCPs on a patient-by-patient basis.

#### Assessment of balance

The HCPs were provided with a copy of the patient’s brief Balance Evaluations and Systems Test (BEST) test scores [29]. Although the researchers conducted detailed balance assessments pre and post-PR to determine the program’s effectiveness, the brief BEST test was chosen rather than supplying the HCPs with the full BEST test because it is quick and easy to interpret. A detailed description of the full BEST test is provided under the heading; ‘balance outcome measures’. The brief BEST test includes just six items, one from each of the sub-systems for balance control (biomechanical, stability limits/verticality, anticipatory postural adjustments for postural transitions, reactive postural response strategies, weighting of sensory information for orientation and postural stability during gait), yet still enables balance training to be tailored according to the specific impaired balance systems identified in individuals patients [29]. Furthermore, HCPs were supplied with a copy of the patient’s brief BEST test scores upon completion of the program to provide individual feedback regarding the efficacy of the training.

#### Content of balance training

For those individuals with COPD enrolled in the study, the first 30 min of each exercise session were dedicated to balance training. Patients would complete a variety of exercises each session which were informed by the results of the brief BEST test. Table 1 displays a sample balance training program. The HCPs recorded patients’ progress in their balance logs and advanced them through the three stages as they saw appropriate.

**Table 1:** Sample Balance Training Program*

Balance exercise stations	Stage 1	Stage 2	Stage 3
Biomechanical	Sit on floor and stand up with chair, lateral leg lifts, heel/toe raises, squats with support.	Sit on floor and stand up without chair, lateral leg lifts with resistance, walking on heels/toes, squats without support.	Sit on floor and stand up holding a medicine ball, side stepping with a resistance, squats with a weight, toe raises on one leg.
Stability limits/verticality	Sitting on a fit ball.	Sitting on a fit ball marching on the spot, sitting on a fit ball and shifting weight from side to side.	Sitting on a fit ball performing leg lifts, sitting on a fit ball whilst throwing and catching a ball.
Anticipatory postural control/transitions	Sit to stand using the chair arms for support, toe taps on a step, arm raises.	Sit to stand without using the chair arms for support, step ups, arm raises with a weight.	Sit to stand with a weight, step ups with a weight, throwing and catching a ball to encourage reaching, step ups and arms raises in combination.
Reactive postural responses	Perturbations in normal stance	Perturbations in narrow stance	Perturbations in tandem stance
Sensory orientation:	Narrow stance eyes closed, tandem stance, normal stance on foam.	Narrow stance on foam with eyes closed, stand on ramp with eyes closed.	Stand on bosu ball, stand on foam whilst throwing and catching a ball.
Postural stability in gait	Walking sideways, walking backwards.	Complete a low level obstacle course.	Complete a high level obstacle course, kick a ball back and forth.

*This program should be refined, adjusted and personalized to the abilities of the individual

#### Staffing

One therapist was required to supervise two patients during the balance training, although this varied depending on patients’ balance ability. Usually, a ratio of one therapist for three patients was adequate but on occasions for some exercises another person was required to supervise. Therefore, in a class size of 12, where six patients are doing balance training alongside PR and six patients are completing standard PR without balance training approximately three physiotherapists were required. Compared to standard PR without balance training one extra physiotherapist was required for 30 min three times a week, translating to an additional 0.05 full time equivalent position.

### Data collection

#### Focus group with the HCPs

Following completion of the study, a focus group, guided by a topic guide and facilitated by a researcher (SH) experienced in qualitative research methods was held with the HCPs to explore their experiences and opinions regarding the feasibility of delivering balance training alongside PR to patients with COPD. The focus group was digitally recorded and transcribed verbatim. Data were analyzed thematically, with the support of NVivo software (v 9; QSR International, Melbourne, Australia). The initial coding for the transcript was conducted by one researcher (SH) and agreed with a second researcher (TA).

#### Descriptive measures for service users

Age, gender, smoking history and pack years, use of walking aids and oxygen usage were recorded. Recent pulmonary function results (Forced Expiratory Volume in one second (FEV_1_) and Forced Expiratory Volume in one second/Forced Vital Capacity (FEV_1_/FVC)) and measurements of height and weight were retrieved from patients’ clinical records.

#### Service users’ evaluation

Patients’ attendance was recorded and adverse events monitored. To measure perceived change in balance status, a global balance transition item was used in which participants were asked to rate the amount of change they experienced in their balance over the training program on a five-point Likert scale (much better, a little better, no change, a little worse, much worse).

Individuals with COPD satisfaction with the training regimen was recorded by adapting an existing questionnaire used in a previous study on KT for single leg cycling in COPD [20]. The questionnaire was completed during patients post-assessment.

#### Balance outcome measures

The balance outcome measures were completed before patients commenced PR and after the six week balance training program by two of three researchers (SH, TA and JR), all of whom had experience administrating the balance tests. The same rater conducted both the pre and post-tests for each individual patient.

The Berg Balance Scale (BBS) [30] consists of 14 items including activities such as: transfers, reaching, turning around and single legged stance. Items are graded on a scale ranging from zero (unable/unsafe) to four (independent/efficient/safe), with higher scores indicating greater balance control. The scale has demonstrated internal consistency, intra-rater and inter-rater reliability, content validity, construct validity and predictive validity for determining falls in older adults [31]. A change of 3.3 (or ≥4) has been suggested to represent the minimal detectable change (MDC) for elderly individuals with baseline BBS scores of 45–56 points. For individuals with lower baseline scores the MDC score is five to six points for community-dwelling older adults [32].

The Balance Evaluation Systems Test (BESTest) [33] evaluates six subsystems of balance control, including; biomechanical, stability limits/verticality, anticipatory postural adjustments for postural transitions, reactive postural response strategies, weighting of sensory information for orientation and postural stability during gait. The BESTest has demonstrated excellent inter-rater reliability and validity, it relates to patients’ balance confidence and it is useful for directing therapy by identifying subscales of balance which are more or less impaired.

The Activities-Specific Balance Confidence (ABC) scale [34] requires patients to indicate their confidence in performing 16 activities without losing their balance or becoming unsteady on an 11 point scale (0-100 %). Higher scores indicate greater balance confidence. The ABC scale has good test-re-test reliability and, internal consistency and predictive capacity for falls in older community-dwelling adults [31, 34]. A change of 13 % has been shown to reflect a MDC in balance confidence in community dwelling older adults.

### Six month follow up

At six months following completion of the research study the researcher (SH) met with the ‘clinical practice lead’ of the PR service to document ‘if’ and ‘how’ balance training was continuing to be implemented within the PR program.

### Statistical analysis

Data were analyzed using the SPSS 22.0 for Windows (SPSS Inc, Chicago, USA). The distribution of data was evaluated using the Shapro-Wilks test and frequency histograms. A Wilcoxon signed ranks test was applied to examine within subject differences in terms of balance measures and a paired *t*-test was used to explore differences in the 6MWT and CRQ-SR pre and post-PR. A bonferroni correction was applied for multiple comparisons (p < 0.01).

A sample size of 19 would yield 80 % power (alpha = 0.05) to detect a difference of four points (MDC)^52^ in BBS before and after the intervention using a paired *t*-test.

## Results

### Participants

All the HCPs who were invited to take part in the study were involved in the delivery of balance training alongside PR including; five physiotherapists, two physiotherapy assistants and one nurse.

### Focus group with health care professionals

Six HCPs attended including; five physiotherapists and one nurse. The nurse and one physiotherapist attended to the out-patient PR program whilst the remaining physiotherapists were involved in the delivery of in-patient PR. The main themes which emerged are provided in Table 2.

**Table 2:** Physiotherapists’ perceptions, barriers and strategies to delivering balance training within Pulmonary Rehabilitation

Themes	Quotes	Key message
Perceptions of balance training within PR	● Benefits	Balance training is effective at improving patients’ balance but distracts from the usual PR program.
	PT4: *“It’s definitely beneficial. I mean you see the difference just through the … through the weeks that we trained them and the patients do notice and they comment that they notice a big difference.”*	
	● Disadvantages	
	PT2: *“I know it’s supposed to be as an adjunct to their normal one* [PR program] *but it actually did impact their normal programme.”*	
	PT1: *“if it’s … some of those sessions are balance where they need supervision. They are not going to be taking home very much* [balance exercises which require supervision] *and you want them to have a good comprehensive home programme.”*	
Barriers to balance training within PR	PT5: *“That’s complicated. Remember those are geriatric patients. Their memories are not that great. Okay? Some they even repeat for weeks still doesn’t stick.”*	Barriers to balance training include:
	PT2: *“We were limited just because first off the … the time restraints. A lot of them* [patients] *wanted to work on their core exercise programmes.”*	1. Time restraints
	PT3: *“if they were in the balance programme sometimes I was putting their sheets in a chart at the end of their … their six week programme and they would have done something [hand therapy] only two or three times the whole time there were here”*	2. Space
	PT1: *“the space, the monitoring both, the group you have in there already plus the close supervision you need to do the balance properly.”*	3. Staffing due to the unpredictability of patients’ balance and patients inability to perform the exercises independently.
	PT3: “*their balance really varied from session to session.”*	
Support for therapists	PT2: *“I think yeah, I mean, it’s nice to see all the equipment that you are planning to use…”*	Support consisting of familiarisation with the equipment and practical demonstrations is necessary for the first few sessions.
	PT3: *“I felt it was helpful that you were there for the first few sessions to kind of get it going in our environment.”*	
	PT2: *“Yeah it’s always good for a … kind of a observation demonstration.”*	
The sustainability of balance training within PR.	PT4: *“Since… sorry, since the study too I tend… I’m more prone to ask them in terms of falls too versus before I wasn’t really, you know, focusing on whether they have previous falls.”*	Aspects of balance training are sustainable but following completion of the study balance training was being delivered fewer times per week.
	PT5: “*And then what we’re doing the TUG and their sit and stand balance……as a part of the assessment.”*	
	PT1: *“I did it with one lady. She gets in and out of a boat so we did some balance exercises and I sent her home with a mini programme.”*	
	PT3: *“We’re not doing balance three times a week. ….as we see there’s a goal of theirs or something that they require”*	
Strategies to improve the sustainability of balance training with PR	PT1: *“Has to be incorporated* [into an interval training program] *cause we don’t have the staff to do it the other way*.”	Strategies to maintain the delivery of balance training include:
	PT2: *“for the lower level people you want to provide the … the one to one for safety in terms of spotting just because their balance is so bad.”*	1. Deliver balance training twice a week.
	PT5:*“You have with the pictures. And that they can do it with parallels* [bars]*”*.	2. Deliver as an interval training program to everyone enrolled in PR.
	PT3: *“you kind of have to educate people to become independent in their exercises.”*	3. Provide visual aids.
	PT5: *“Have to be simple…Functional. And easy.”*	4. Promote independence by providing a set program consisting of simple balance exercises.
	PT3: *“if we had an extra person like if there were no follow ups or something we had… we had extra help, then we would have that extra person just to deal with the other patients”*	5. Consider the environment (i.e. use of parallel bars) if staffing is not available.
	PT3: *“And so when we changed the sheets I found that was a … at least cause I knew had a sort of a set programme and then the primary therapist could progress it as they wanted to.”*	6. Introduce a home-based program early.
	PT3: *“where everyone is doing it and its circuit based I think is one thing versus just have it incorporated into the IT* [interval training] *programme and then they do it whenever they’re doing their IT.”*	
	PT4 *“balance class and having other people within the room doing exercise it gets chaotic so it’s just having two sets of balance class where everybody does balance and everybody has their own programmes we can have a bit more supervise from the staff”*	
	PT2: *“you’d have to really focus in on where their impairment is.”*	
	PT2: *“maybe it’s advantageous to pick … pick early on which ones are you going to be doing while you… when they go home.”*	

### Service users

**Fig 1.**
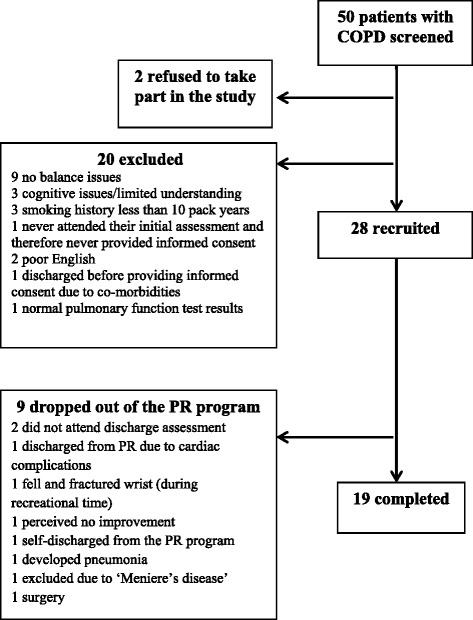
Recruitment flow diagram

Fifty patients with COPD were screened and 28 were recruited to the study. Two patients refused to take part and 20 were excluded with reasons documented in Fig. 1. Of the 28 patients recruited, nine dropped out of the PR program, although balance training was never cited as a reason for patient drop out. In total, 19 patients completed the study, of these 16 were enrolled in the in-patient PR program and three attended the out-patient PR program. Demographic information for the 19 individuals with COPD appears in Table 3.

**Table 3:** Patient characteristics (n = 19)

Demographics	Mean	SD
Age	73	6
Males (n)	10	
BMI	28	8
FEV_1_ % pr	41	17
FEV_1_/FVC	41	16
Pack years (Median (IQR)	50	40-80
Gait aid (n)	13	
Oxygen (n)	7	

SD = standard deviation; BMI = body mass index; FEV_1_% pr = forced expiratory volume in one second, percent predicted; FEV_1_/FVC forced expiratory volume in one second/forced vital capacity; Interquartile range (IQR)

Patients completed 12 (range: 10–14) out of a possible 18 balance training sessions and no adverse events were reported. Forty seven percent (n = 9) of patients perceived their balance to be “much better” after training, 47 % (n = 9) perceived their balance as “a little better” and 5 % (n = 1) of patients reported no change.

According to the satisfaction survey, 84 % of patients reported that balance training helped them with everyday activities, 84 % enjoyed balance training, 95 % of patients said they would recommend balance training for other people with COPD and 95 % indicated their wish to continue with it.

### Changes in outcome measures

Table 4 shows within-group changes for measures of balance performed pre and post the training program. These results are also compared with measures of the same balance intervention obtained during the efficacy study [24].

**Table 4:** Within-group changes in measures of balance compared to the recent RCT [24] (mean (SD))

	KT trial			RCT trial		
Outcome	Pre-test	Post-test	P value #	Pre-test	Post-test	P value
BBS	46.3 (7.1)	53.3 (3.7)	p < 0.001	45.6 (5.8)	52.6 (2.9)	p < 0.001
BESTest – biomechanics	8.1 (3.2)	10.6 (2.9)	p = 0.001	9.4 (2.6)	11.9 (2.5)	p < 0.001
BESTest – stability	14.5 (2.4)	17.6 (1.1)	p < 0.001	16.4 (2.4)	17.8 (1.7)	p = 0.001
BESTest – transitions	11.5 (3.3)	14.4 (2.9)	p < 0.001	12.6 (2.9)	14.2 (2.4)	p = 0.001
BESTest – reactive	12.6 (3.7)	15.6 (2.3)	p = 0.001	13.3 (4.0)	15.4 (2.4)	p = 0.006
BESTest – sensory	10.5 (2.6)	13.4 (2.3)	p < 0.001	11.5 (2.1)	13.2 (1.4)	p < 0.001
BESTest – gait	9.7 (4.3)	15.0 (4.8)	p < 0.001	11.6 (4.0)	14.4 (3.2)	p < 0.001
BESTest total	66.9 (15.2)	86.6 (12.3)	p < 0.001	66.4 (13.1)	82.0 (9.2)	p < 0.001
ABC score	69.2 (24.9)	78.2 (17.3)	p = 0.005	57.6 (24.0)	74.6 (13.0)	p < 0.001

PR = pulmonary rehabilitation; BBS = Berg balance test; BESTest = Balance evaluations and systems test; ABC = activities-specific balance confidence

# = *non-parametric tests applied*

Following PR with balance training improvements were noted in: the 6WMT (mean difference (SD)) 63 m (35.9) and all domains of the CRQ-SR: dyspnea 1.9 (0.2), fatigue 1.7 (0.3), emotion 1.6 (0.4) and mastery 1.9 (0.4) which were both clinically and statistically significant (p < 0.001).

### Six month follow up

Without support from the researchers, the HCPs are continuing to deliver balance training twice a week to all patients enrolled in PR at WPHC. The brief BEST test and the ABC confidence scale is being completed as part of the pre and post-assessment for PR. HCPs are prescribing balance exercises for patients as part of their home-based exercise program, patients are introduced to these exercises in week one of the program.

## Discussion

This study adds to the limited body of evidence reporting on KT interventions delivered within a rehabilitation setting, narrowing the gap which exists between evidence and clinical practice. The findings highlight the feasibility, effectiveness and sustainability of implementing balance training into PR programs for patients with COPD. HCPs were able to identify a number of strategies to improve the ease of delivering balance training alongside PR which could serve to inform the implementation of other interventions into rehabilitation settings. Balance training with PR was readily accepted by patients who reported finding the training enjoyable and beneficial in terms of their everyday activities.

The purpose of this study was to translate knowledge gleaned from clinical research into the clinical service at WPHC [8, 22–24]. We adopted a practical approach to KT, utilizing our strong relationship with the clinical team. Although, our approach to KT was not theoretically based, theories do support what we did. For example, the Theory of Planned Behavior suggests that an individual’s intentions to adopt an intervention can be determined by feelings of control [35]. By involving the HCPs in the development of the program, feelings of ‘ownership’ were elicited and all testified to the benefits of balance training for patients with COPD. This positive view may, in part, have been attributed to the feedback provided in the form of patients’ pre and post brief BESTest scores which served not only to inform the content of the program but to demonstrate areas of balance on which the training had an effect. Feedback was provided individually to the HCPs in keeping with the recommendation put forth by the Feedback Intervention Theory [36]. Providing individual feedback can avoid social comparison with peers potentially threatening feelings of self-efficacy.

Despite recognizing the benefits of balance training at improving measures of balance control associated with a risk of falls, HCPs initially expressed negative attitudes regarding the sustainability of balance training. However, when prompted, HCPs were able to identify a number of strategies to improve the ease of delivering balance training, including reducing the number of training sessions to twice a week (which deviates slightly from the evidence on the effectiveness of balance training in patients with COPD [24]). Determining the optimal number of balance training sessions to achieve a meaningful improvement in balance and fall risk required would be an important area for future research. Furthermore, HCPs found it easier to adopt a ‘blanket approach’ to balance training. Although delivering balance training to everyone enrolled in the PR program may not be considered a ‘lean’ and efficient use of resources, we do not yet know which patients would be most likely to benefit from balance training. Also, when asked, the majority of patients (82 % in our current study and 93 % in our previous RCT [24]) were identified as having an increased falls risk when asked the question: “have you had a fall in the last five years or a recent near fall?”. This is not surprising considering patients with COPD are often elderly, frail and exhibit high levels of inactivity.

Balance training delivered with PR as part of a clinical service resulted in improvements for all measures of balance and falls risk whilst eliciting significant and clinically important changes in standard PR outcomes. There was considerable overlap between usual PR lower limb strength training and certain balance exercises (biomechanical and anticipatory postural control/transitions). Some balance training was also likely to challenge patients aerobically, for example, postural transitions in gait. This may provide an explanation for why balance training did not impact other health outcomes traditionally associated with PR, despite spending less time completing the traditional aerobic and strength training included in PR.

Balance training was well received by patients. These results are in line with those from our previous study where an arguably more intensive balance training program was delivered by research staff with a lower staff to patient ratio and isolated from the clinical PR service (Table 4) [24]. Currently, the optimum approach (dose, duration, setting) for balance training is unknown and remains to be determined.

The findings presented in this paper are based on our experience implementing balance training within a specialist rehabilitation hospital where the health care professionals had some prior knowledge of balance deficits and the effectiveness of balance training in individuals with COPD. The ability to implement balance training in a general hospital-based PR program is still unknown and is likely to offer additional challenges in terms of buy-in, staffing and time restraints. However, the model described is one that was effective and could be used as a template to develop a conceptual model for KT in PR which could be applied in other centers (Additional file 1). The inclusion of patients enrolled in both in-patient and out-patient PR programs extends the relevance of findings to both modes of PR delivery; however, there were few subjects included from the out-patient program and the majority of HCPs who participated in the focus group were involved in the delivery of in-patient PR. More studies are likely required to inform how to successfully incorporate balance training into an out-patient PR settings with less frequent exercise sessions. Collecting measures of balance control via a clinical audit at six months would have been a stronger way of assessing the sustainability of the program and could be conducted in the future.

## Conclusions

The delivery of balance training within PR is feasible, effective and sustainable for patients with COPD. By successfully translating the lessons learnt from efficacy studies of balance training into a sustainable clinical service we have minimized the gap which currently exists between evidence and clinical practice. This study also contributes to the limited body of evidence reporting on KT projects conducted within in the area of rehabilitation.

## Additional file

Additional file 1:
**Table outlining the KT actions and subsequent effect.**

## Abbreviations

ABC: Activities-specific balance confidence
BEST: Balance evaluations and systems test
BBS: Berg balance scale
BMI: Body mass index
COPD: Chronic obstructive pulmonary disease
CRQ-SR: Chronic respiratory questionnaire-self-reported
FEV_1_: Forced expiratory volume in one second
FEV_1_/FVC: Forced expiratory volume in one second /forced vital capacity
HCPs: Health care professionals
KT: Knowledge translation
MDC: Minimal detectable change
PR: Pulmonary rehabilitation
RCT: Randomized controlled trial
6MWT: Six-minute walk test
SD: Standard deviation
WPHC: West park healthcare center
