# Supplementary material for: Minimizing the evidence-practice gap – a prospective cohort study incorporating balance training into pulmonary rehabilitation for individuals with chronic obstructive pulmonary disease
Source: BMC Pulm Med. 2015 Jul 23;15:73. doi: 10.1186/s12890-015-0067-2 (PMC4511452; doi:10.1186/s12890-015-0067-2)
Supplement: Additional file 1: — Table outlining the KT actions and subsequent effect. [file 12890_2015_67_MOESM1_ESM.pdf]

Additional file 1: Table outlining the KT actions and subsequent effect

	**Knowledge Translation action**	**Knowledge Translation effect**
**Step 1**	One hour training session regarding the benefits and practical applications of balance training and familiarization with the balance equipment	Increased knowledge and awareness of the benefits of balance training
**Step 2**	Assistance with the balance training sessions delivering guidance on; prescription, progression and providing practical demonstrations as requested	Increased trust and rapport with the health care professionals
**Step 3**	Program development, led by the health care professionals responsible for delivering pulmonary rehabilitation	Increased feelings of ownership and likelihood of sustainability
**Step 4**	Development of program logs, led by the health care professionals responsible for delivering pulmonary rehabilitation	Increased feelings of ownership and likelihood of sustainability
**Step 5**	Brief BEST tests results pre and post pulmonary rehabilitation are provided on a one-to-one basis.	Refection on performance and enhanced motivation
